# Age-specific influences of refractive error and illuminance on pupil diameter

**DOI:** 10.1097/MD.0000000000029859

**Published:** 2022-07-08

**Authors:** Yong-Sik Lee, Hui-June Kim, Dong-Kyu Lim, Myoung-Hee Kim, Koon-Ja Lee

**Affiliations:** a Department of Optometry, Eulji University, Seongnam-si, Gyeonggi-do, Korea; b Department of Optometry, GM St. Mary’s Eye Clinic 2, Saessak-ro, Busanjin-gu, Busan-si, Korea; c Department of Dental Hygiene, College of Health Science, Eulji University, Seongnam-si, Gyeonggi-do, Korea.

**Keywords:** age, illuminance, pupil diameter, refractive error

## Abstract

To assess the most influential factor for pupil diameter changes among age, illuminance, and refractive state and reestablish the optimal procedures for clinical applications based on refractive state and illuminance for different age groups.

The study was an observational study (repeated measure study). Participants included 219 Korean adults aged 20 to 69 years. Pupil diameters were measured using a pupilometer under scotopic, mesopic-low, and mesopic-high lighting conditions. Factor interactions among age, illuminance, and refractive state were evaluated using mixed linear model and chi-square automated interaction detection.

Illuminance mainly contributed to variations in pupil diameter of participants over 50 years, whereas the refractive state was the dominant controlling factor for the pupil variation in participants below 50 years. For more generalized application, the pupil diameter decreased with older age and brighter illuminance (*P* < .001, inverse correlation, all comparisons). The mean pupil diameter was significantly higher in myopes and emmetropes than in hyperopes (*P* < .001). Pupil diameter variation modeled using the mixed model confirmed age, illuminance, and refractive error as significant factors (*P* < .001).

Accounting for the interactions among age, illuminance, and refractive error and establishing their hierarchical dominance can be generalized using the chi-square automated interaction detection method and mixed model. Promoting age-dependent consideration for both illuminance and refractive state is necessary when pupil diameters play significant roles in clinical and manufacturing circumstances.

## 1. Introduction

Pupil size plays a crucial role in improving visual performances in response to biological and environmental factors. Since the 1950s, a growing request for reliable information on pupil control regulating factors has led researchers to evaluate the influence of factors, such as luminance and age. However, until recently, the role of refractive state or error in pupil variation has been controversial. According to some studies, the most important variable controlling pupil diameter is the light reflex.^[1,2]^ Using 222 subjects aged 20 to 89 years, 1 study reported a nonlinear age-dependent decrement in pupil size, although this study lacked illuminance control.^[3]^ Using various illuminance settings, another study demonstrated that pupil sizes decrease linearly with age using 91 individuals aged 17 to 83 years.^[4]^ Several studies have suggested an inverse correlation between age and pupil size in preoperative refractive surgery patients under varying luminance conditions.^[5,6]^

Additional studies have attempted to identify other contributing factors, such as refractive error, sex, or iris color. However, there has been convincing evidence against any correlations among pupil size, sex, and iris color.^[4–8]^ Most previous studies relied on statistical methods that compared mean pupil size differences while ignoring the relative influences of contributing factors. Meanwhile, many studies have proven that the effects of refractive errors on pupil sizes are significant. One of such studies reported smaller pupil sizes in hyperopes than in myopes among 266 male university students.^[9]^ In 1990, a study found no difference in pupil size between low myopes and emmetropes; however, that study included only young individuals (18–26 years).^[8]^

In 1994, another study also examined interactions between refractive state and pupil size and detected no significant relationships in myopes, emmetropes, and hyperopes.^[4]^ However, the 10-year age difference between older hyperopes and younger myopes could represent a biasing factor. Only 1 study^[4]^ may have had the adequate statistical power to perform subanalyses and interaction analyses, but a significant limitation of this study strengthens the rationale for the present study. Although no relationship between pupil size and refractive state was detected in a relatively small sample of patients undergoing refractive surgery,^[5]^ larger studies with refractive surgery candidates concluded that the preoperative refractive state affects pupil size under mesopic conditions, with smaller pupil sizes in hyperopes than myopes.^[6]^

The performance of multifocal contact lenses also depends on pupil size, and pupil performance should be evaluated at various viewing distances and luminance levels.^[10–13]^ By evaluating the relationship between pupil diameters and factors like age, illuminance, and refractive state in an adult Korean population, we aim to discover more localized forms of information that could be applied for contact lens design and clinical situations such as planning and outcome of refractive surgery, anisocoria, and neuro-ophthalmological investigations.^[14–16]^

## 2. Methods

### 2.1. Study population and measurements

Korean adults (20–69 years) were enrolled, excluding participants with ophthalmological disorders, for example, diabetes mellitus or extreme cases of myopia/hyperopia. This descriptive case series study was approved by the Research Ethics Committee of the Graduate School of Medicine and Faculty of Medicine at the University of Eulji (EU17-32) and performed according to the Declaration of Helsinki. All participants provided written informed consent.

The NIDEK autorefractorkeratometer (NIDEK Inc., Fremont, CA) was used for objective refraction assessment in all participants. Ophthalmological disorders were screened by ophthalmologists. Pupil diameters were measured using the pupilometer VIP-200 (NEUOPTICS, Irvine, CA), widely used in clinical practice and academic research. A concealing gasket protected measurement from light contaminants, while the laboratory illumination was kept at 100 lx. To prevent interference by accommodation during diameter measurements, the consensual eye aimed at an object at a distance of 5 m. The light settings simulated real-world conditions: Scotopic, a pitch-dark environment, mesopic-low nighttime driving, and mesopic-high dawn or night illumination. For example, “the variation in pupil diameter is most marked at low luminance, the influence of the pupil is critical for medium and low luminance performance.”^[2]^ Also, the significant effect of low and medium luminance on pupil size in real-life cognitive tasks has been reported.^[17]^ It has been found that “effect of cognitive arousal on pupil size interacts multiplicatively with luminance, with the largest effects occurring at low and medium luminance.”^[18]^

### 2.2. Statistical models

The overall distributions of refractive errors (spherical equivalent) and pupil diameter were examined according to 4 age groups and 3 types of refractive error. The mean and the standard deviation for the pupil diameter were presented. By stratifying the 3 types of illuminance, pupil diameters were summarized according to 4 age groups and 3 types of refractive error. One-way analysis of variance was used for the mean difference in each age group and illuminance condition. In addition, by layering age groups, the pattern of pupil diameters according to illuminance condition was examined as graphics.

Finally, in order to quantify the impact of factors such as age, illuminance on the average pupil diameter we used the linear mixed model.^[19–21]^ To estimate the model coefficients, penalized quasi-likelihood methods based on restricted or residual maximum likelihood were used. The results of the mixed model are reported in the form of effect estimates and its confidence interval in Table 3.^[18,22]^

For this analysis, pupil diameter was considered a dependent variable. Independent factors were age groups (20–39 years; 40–49 years; 50–59 years; and 60–69 years), illuminance condition (scotopic: 0.0 lx; mesopic low: 0.3 lx; mesopic high: 3 lx), refractive error (best sphere: myopes, ≤–0.5 D; emmetropes, ≥–0.25 to ≤+0.50 D; and hyperopes, ≥+0.75 D), and sex.

In order to determine the most influencing factors, a chi-square automated interaction detection (CHAID) was performed.^[2,23]^ The CHAID analysis is popular as it not only finds association between the response variable and the independent factors but also the significance of the interactions between them quantified via chi-statistic (for categorical response variables) and F-statistics (for continuous response variables) and their associated *P* values. Being nonparametric, CHAID is preferred as it does not require estimation as in linear regression. The criterion used by the CHAID analysis for determining the hierarchy of the significance level is the *P* value of the F statistic (as our response variable, pupil diameter, is numerical) of the mean group difference.^[24,25]^ For each split in the CHAID, an *F* value is calculated based on mean difference between levels of an independent (categorical) variable with the pupil diameter. The collection of *P* values corresponding to the F statistic (with a given degree of freedom) forms a set. The independent variable corresponding to the least *P* value is deemed the one with the highest significance in that split. This process is repeated until all the independent variables are exhausted.

Data were analyzed using R software version 4.0.2 (Comprehensive R Archive Network: http://cran.r-project.org). In all analyses, a *P* value <.05 was considered statistically significant.

## 3. Results

### 3.1. Subject characteristics of the age, refractive state, and pupil size

In Table 1, the mean pupillary diameter and mean refractive error according to age groups are provided. The average reflective state of emmetropes, hyperopes, and myopes in an entire cohort of 219 subjects (mean age of 52.70 years) respectively showed +0.11 D, +1.51 D, and –2.01 D. The average pupil size of emmetropes, hyperopes, and myopes were 5.36, 5.06, and 5.45 mm, respectively.

**Table 1:** Baseline characteristics according to age group and refractive error group.

	Total	Aged 20–39 yr (n = 39)	Aged 40–49 yr (n = 33)	Aged 50–59 yr (n = 59)	Aged 60–69 yr (n = 88)
Characteristics	Mean (SD)	Mean (SD)	Mean (SD)	Mean (SD)	Mean (SD)
Age (yr)	52.70 (13.10)	29.18 (5.00)	45.48 (2.76)	55.34 (2.56)	64.14 (2.66)
Spherical refractive error (diopter)					
Emmetropes	0.11 (0.26)	0.02 (0.23)	–0.06 (0.18)	0.16 (0.28)	0.17 (0.25)
Hyperopes	1.49 (0.70)	1.04 (0.37)	1.06 (0.31)	1.43 (0.59)	1.60 (0.76)
Myopes	–2.01 (1.58)	–1.27 (0.94)	–2.28 (1.70)	–2.36 (1.68)	–1.84 (1.48)
Pupil diameter (mm)					
Emmetropes	5.36 (0.76)	5.69 (0.66)	5.79 (0.79)	5.39 (0.73)	5.04 (0.70)
Hyperopes	5.06 (0.73)	4.32 (0.47)	5.12 (0.45)	5.23 (0.74)	5.08 (0.73)
Myopes	5.45 (0.89)	5.80 (0.89)	5.69 (0.72)	5.08 (0.94)	5.26 (0.77)

Values presented as mean ± SD for continuous variables.

SD = standard deviation.

### 3.2. Characteristics of the pupil variation for the interaction among age, illuminance, and refractive state

**Figure 1.**
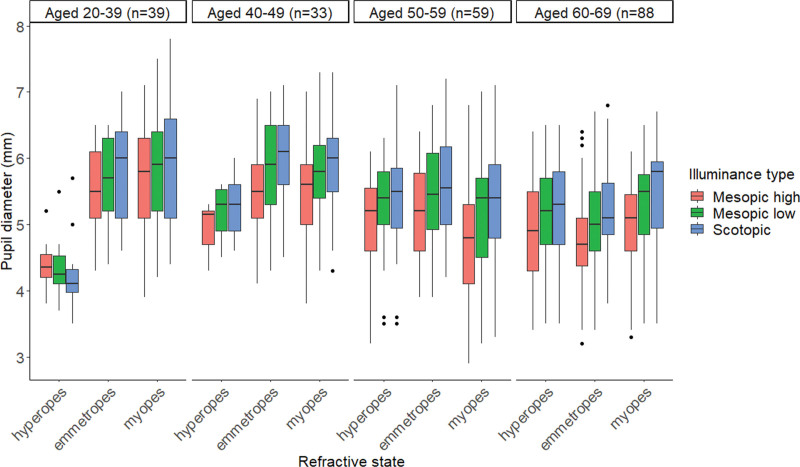
Pupil diameter (mm) as a function of age for each refractive error group at the 3 illuminance levels (scotopic 0.0 lx, mesopic low 0.3 lx, and mesopic high 3.0 lx) tested. Age ranges in years: Aged 20–39 yr; Aged 40–49 yr; Aged 50–59 yr; and Aged 60–69 yr.

In Table 2 and Figure 1, the mean pupil sizes are indicated according to illuminance, age, and refractive error. Together with the conventional literature, our results indicated decreased pupil diameters as the illuminance level increased (Table 2). As a matter of fact, when the illuminance intervened in the interaction between age and refractive state, the refractive state had a significant effect on the young age group <40 years, that is “aged 20 to 39” group showed significant value (scotopic, mesopic low, and mesopic high; *P* < .001). However, the effect of the refractive state was only marginally significant for older ages >40 years (*P* ≥ .05) except for the age group “aged 40 to 49” (scotopic; *P* < .05).

**Table 2:** Pupil diameter of illuminance stratification according to age group and refractive error group.

	Total	Aged 20–39 yr (n = 39)	Aged 40–49 yr (n = 33)	Aged 50–59 yr (n = 59)	Aged 60–69 yr (n = 88)
Illuminance	Mean (SD)	Mean (SD)	Mean (SD)	Mean (SD)	Mean (SD)
Scotopic					
Emmetropes	5.54 (0.75)	5.83 (0.72)	6.02 (0.76)	5.56 (0.70)	5.24 (0.69)
Hyperopes	5.19 (0.76)	4.40 (0.80)	5.25 (0.50)	5.41 (0.74)	5.22 (0.71)
Myopes	5.64 (0.86)	5.90 (0.93)	5.89 (0.68)	5.31 (0.89)	5.49 (0.78)
*P* value	<.001	<.001	.034	.384	.219
Mesopic low					
Emmetropes	5.41 (0.73)	5.72 (0.61)	5.88 (0.78)	5.45 (0.70)	5.09 (0.68)
Hyperopes	5.13 (0.71)	4.34 (0.46)	5.18 (0.44)	5.32 (0.67)	5.15 (0.71)
Myopes	5.51 (0.87)	5.85 (0.84)	5.76 (0.69)	5.15 (0.94)	5.31 (0.76)
*P* value	<.001	<.001	.058	.237	.411
Mesopic high					
Emmetropes	5.12 (0.75)	5.51 (0.62)	5.48 (0.78)	5.16 (0.73)	4.79 (0.67)
Hyperopes	4.86 (0.70)	4.39 (0.37)	4.94 (0.39)	4.98 (0.75)	4.87 (0.71)
Myopes	5.19 (0.90)	5.66 (0.89)	5.42 (0.72)	4.78 (0.94)	4.98 (0.71)
*P* value	<.001	<.001	.173	.121	.486

All persons (N = 221).

Values presented as mean ± SD for continuous variables.

*P* value is calculated as analysis of variance.

SD = standard deviation.

### 3.3. Mixed linear model

Using the mixed linear model with repeated measures, the predicted pupil variations were produced based on each controlled variable, as shown in Table 3. For age groups, the most significant variation in pupil diameter occurred in the “aged 40 to 49” group (–0.319 mm, *P* < .001) compared to the “aged 20 to 39” group. Moreover, the “aged 50 to 59” and “aged 60 to 69” groups showed pupil variations of –0.088 mm (*P* < .1) and 0.182 mm (*P* < .001), respectively. The illuminance levels of each mesopic low and high, respectively, altered pupil diameters by –0.105 mm (*P* < .05) and –0.397 mm (*P* < .001) compared to the scotopic condition. The best spheres for myopes and hyperopes, respectively, indicated pupil variations of 0.004 mm (*P* > .05) and –0.224 mm (*P* < .001) compared to emmetropes. Meanwhile, sex does not significantly contribute to pupil variation.

**Table 3:** Effect estimates from the linear mixed model of changes in average pupil diameter in 219 participants with repeated measures of the 3 types of illuminance.

	Estimates	95% CI
	Pupil diameter (mm)	
Age group (yr)		
20–39	Reference	
40–49	–0.319***	–0.409 to –0.229
50–59	–0.088	–0.180 to 0.005
60–69	0.182***	0.088–0.277
Sex		
Male	Reference	
Female	–0.018	–0.107 to 0.070
Illuminance (lx)		
Scotopic (0.0)	Reference	
Mesopic low (0.3)	–0.105*	–0.207 to –0.003
Mesopic high (3)	–0.397***	–0.499 to –0.295
Best sphere (diopter)		
Emmetropes (≥–0.25 to ≤+0.50)	Ref	
Hyperopes (≥+0.75)	–0.224***	–0.330, to –0.117
Myopes (≤–0.5)	0.004	–0.103 to –0.110

CI = confidence interval.

A *P* value significance codes:

* 0.05,

**0.01,

*** 0.001.

### 3.4. CHAID analysis

**Figure 2.**
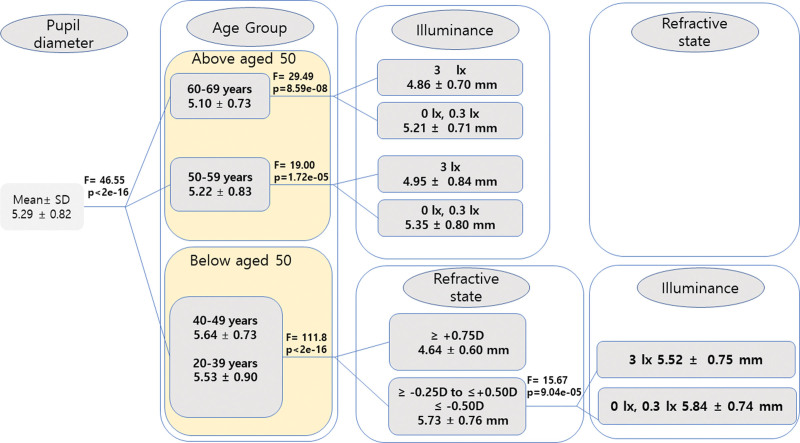
CHAID tree diagram for the analysis of pupil diameter (dependent variable). Three independent variables, age, refractive state, and illuminance, are tested against the dependent variable during the branch split and 1 variable with the minimum *P* value is selected. The selected variables (or its levels) with the *P* and *F* values are shown. Varying degrees of pupil diameter (in mm) are shown based on their significance levels; the factors are differentiated forward through each branch based on their significance levels assessed using *F* values. The first level represents age as the most dominant factor (highest significance level), followed by illuminance (second-highest level of significance) and the least significant refractive state in participants over 50 yr of age. For participants under 50 yr of age, refractive state is the second-highest level of significance, followed by the least significant variable, illuminance. CHAID = chi-square automated interaction detection.

As depicted in Figure 2, age (*P* < .001, *F* = 46.55) was the most influencing factor affecting pupil size and thus appeared as the first variable in the tree. Among the age groups, CHAID was found for the age >50 years (“aged 50–59” and “aged 60–69”) the illuminance was the next influencing factor (*P* < .001); the refractive state having no significant influence in these age groups. In the “aged 20 to 39” and “aged 40 to 49” groups, the second most significant influencing factor was the refractive state followed by the illuminance. Thus, we observed from our data that the order of the influencing factors differs between the age groups. Because the CHAID splits the data based on the age group, we provide in Table 4 the description of pupil diameter along with group significance using analysis of variance separately for age group ≥50 years and age group <50 years.

**Table 4:** Pupil diameter of illuminance stratification according to age group and refractive error group.

	Total	Aged <50 yr (n = 72)	Aged ≥50 yr (n = 147)
Illuminance	Mean (SD)	Mean (SD)	Mean (SD)
Scotopic			
Emmetropes	5.54 (0.75)	5.89 (0.73)	5.36 (0.71)
Hyperopes	5.19 (0.76)	4.64 (0.74)	5.27 (0.72)
Myopes	5.64 (0.86)	5.89 (0.80)	5.38 (0.85)
*P* value	<.001	<.001	.533
Mesopic low			
Emmetropes	5.41 (0.73)	5.77 (0.66)	5.23 (0.71)
Hyperopes	5.13 (0.71)	4.68 (0.61)	5.20 (0.70)
Myopes	5.51 (0.87)	5.80 (0.75)	5.21 (0.88)
*P* value	<.001	<.001	.954
Mesopic high			
Emmetropes	5.12 (0.75)	5.50 (0.66)	4.93 (0.72)
Hyperopes	4.86 (0.70)	4.61 (0.46)	4.90 (0.72)
Myopes	5.19 (0.90)	5.52 (0.80)	4.85 (0.87)
*P* value	.004	<.001	.811

Values presented as mean ± SD for continuous variables.

*P* value is calculated as analysis of variance.

SD = standard deviation.

**Figure 3.**
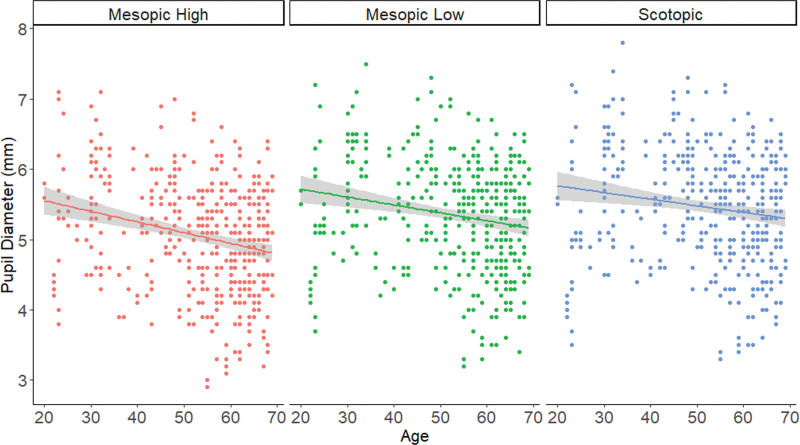
Plot of pupil diameter vs age as a function of illuminance levels. In all levels, as the age increases and as the illuminance of the light get brighter, the pupil diameter decreases (inverse correlation). We confirm this effect also by linear regressing per illuminance level and the results are indicated in Table 5.

All age groups differed significantly in pupil diameter with an inverse age correlation, that is, a decreasing pupil size with increasing age. Figure 3 indicates the plot of pupil diameter as function of age and illuminance levels. We observed that as the age increases and as the illuminance of the light gets brighter, the pupil diameter decreases. To confirm this inverse relationship, we also applied linear regression per illuminance level and the results are indicated in Table 5. The significant increase in the negative correlation is evident in Table 5.

**Table 5:** Results of linear regression of age on pupil diameter for various light illuminance.

Light illuminance	Age	Intercept
Mesopic high	–0.015***	5.858***
Mesopic low	–0.011**	5.940***
Scotopic	–0.009**	5.951***

*P* value significance codes.

*0.05,

** 0.01

*** 0.001.

## 4. Discussion

In this study, we aimed to clearly understand the order of the factors that influence pupil diameter. Using the CHAID analysis widely used for obtaining the hierarchy of influencing factors, we found that the primary factor is the age group (Fig. 2), which aligns with the results of the previous studies.^[26]^ Within the age groups, we found striking evidence that for an age group >50 years of age, illuminance was the only factor that influences the pupil diameter. This indicates that the mid-to-old age groups are susceptible to sudden lighting alterations, such as scotopic or mesopic-low environments changing to mesopic-high conditions, for example, a car approaching at night. The significant pupil size variation between mesopic-low and mesopic-high conditions supports this statement, especially in people over 50 years of age wearing multifocal contact lenses of pupil-dependent optical design. In contrast, for the age group <50 years of age, the order of the influence was found to be a refractive state followed by illuminance. This order is important as it has been found that younger patients and patients with high myopia and/or with the rule astigmatism deserve a more strict and cautious preoperative evaluation of refractive state to decrease postoperative night vision complaints.^[27]^

Previous studies^[28,29]^ have assessed factors for pupil variation. The common determinants of recent studies were age and refractive error. They found that smaller pupils with older age and in hyperopes than in myopes.^[6,7]^ However, the studies did not confirm refractive error as a factor controlling pupil diameter,^[4,28]^mainly due to small sample sizes preventing meaningful estimations. Moreover, a limited source of the high photopic range corresponding to daylight settings to estimate illuminance has been studied.^[4,28]^ Our study overcame these limitations and determined pupil variations throughout various lighting conditions to establish the optimal parameters for providing a more generalized form of medical information.

Several studies indicate that the performance of multifocal and bifocal contact lenses relies heavily on pupil size. Those contact lenses offer focused and defocused images on the retina simultaneously, and the balanced performance between near and distance vision should be considered under various lighting conditions.^[2,10–13,28–30]^ Our results suggest customized lens designs and clinical approaches involving steeper progressions for distant-to-near corrections in people over 50 years, hyperopes, and professionals.^[2,30]^

The investigation on factors influencing pupil size under varying lighting conditions suggested that factors like adapting field size, aging, single vision, and binocular vision play major roles even if individual differences could have confounding effects.^[29]^ Therefore, rather than reaching premature conclusions on pupil variation solely based on age, illuminance, and refractive state, it is also necessary to make careful estimates of other confounders like intelligence and structural eye integrity. Structural eye integrity-associated factors have been shown to influence pupil variations, and as significant factors, logMAR best-corrected visual acuity, mean retinal nerve fiber layer thickness, central corneal thickness, and spherical equivalent have been described.^[31]^ Moreover, cognitive processes, like fluid intelligence more than working memory capacity, cause larger pupil sizes.^[32]^ One review reported that in the 3 cognitive control domains of updating, switching, and inhibition, a higher task demand resulted in increased pupil dilation.^[17]^ Moreover, an original research study demonstrated an increase in pupil diameter with Raven’s Advanced Progressive Matrices fluid intelligence test for the exploration process during analogical reasoning, which coincides with prominent theories of locus coeruleus norepinephrine function.^[33]^ All those pupil-altering factors should be collectively considered to comprehend the dynamics of pupil variation and accordingly apply them rightly. Finally, pupil size after mydriasis is genetically determined with up to 80% heritability.^[34]^ The fact that in a similar study of western countries, illuminance is a predominant factor for pupil variation^[2]^ contradicts our results that age is the most contributing factor.^[2]^ It may be attributed to our population characteristics. Future studies should examine whether Korean adults differ from western populations in this respect.

Regarding study limitations, we could not consider the factor accommodation despite its known effects on pupil diameter. The etiological explanation for myopes and emmetropes having larger pupils than hyperopes can be described by understanding the relationship between pupil magnification and the chief ray angle, resulting in the larger appearance of the pupil than the iris. The entrance pupil, measured in every clinical setting, is the virtual image of the real pupil produced by the cornea and is known to be placed at approximately 0.5 mm in front of the real pupil in the 14% magnified form.^[35]^

Pupil magnification has been defined as “the ratio of the paraxial exit-pupil diameter to the paraxial entrance-pupil diameter,” and the entrance pupil has been defined as the “image of the stop seen through the elements preceding it is.”^[36,37]^ Hence, we strongly believe that myopes, influenced by the different corneal power (higher) and shape (steeper), have a larger pupil (entrance pupil image) diameter than hyperopes with less corneal power and shape.

From a gerontological perspective, with aging, there is a report of the quantitively decreased area of retinal ganglion cell dendritic and axonal arbors, resulting in decreased coverage of the visual field accompanied by the decrease in density of cells and synapses.^[38]^ This report supports our results that pupil variations were mainly affected by refractive state, which is related only to the age group <50 years. Meanwhile, why pupil variation is affected only by illuminance^[39]^ and not refractive state in subjects >50 years old remains unclear. It would be a good starting point to look at qualitative features of retinal connectivity since the study specifies the sustained functionality of molecular identity, laminar specificity of arbors in the inner plexiform layer, and complex responses of retinal ganglion cells to visual stimuli, regardless of the aging process.^[38]^

For the main effect of age, refractive state and illuminance were significant contributing factors for pupil diameter changes. The CHAID model specified their validity in terms of interaction among pupil size controlling factors, especially age, the most dominant factor. The refractive state was significantly associated with greater pupil sizes in myopes and emmetropes than in hyperopes, and the viable reason was explicitly discussed in the etiological perspectives, illustrated by the relation between pupil magnification and chief ray angle. Future studies of neurological approaches, including natural pairings based on morphological and physiological similarities and differences, can facilitate a more profound understanding of the visual system and its proper applications in various clinical situations.

## Acknowledgments

We express our utmost thanks to those who participated in this study.

## Author contributions

Conceptualization: Yong-Sik Lee, Hui-June Kim, Dong-Kyu Lim, Myoung-Hee Kim, Koon-Ja Lee.

Data curation: Yong-Sik Lee, Hui-June Kim, Dong-Kyu Lim

Formal analysis: Hui-June Kim, Dong-Kyu Lim.

Methodology: Yong-Sik Lee, Myoung-Hee Kim, Koon-Ja Lee.

Project administration: Koon-Ja Lee.

Supervision: Koon-Ja Lee.

Validation: Myoung-Hee Kim.

Writing – original draft: Yong-Sik Lee, Koon-Ja Lee.

Writing – review & editing: Yong-Sik Lee, Koon-Ja Lee.
